# In This Issue

**Published:** 2014

## MEASURING THE BURDEN: ALCOHOL’S EVOLVING IMPACT

Alcohol’s impact stretches far beyond the drinker, affecting his or her family as well as the community at large. This widespread impact makes alcohol a major public health concern. This introductory article by Drs. Ralph Hingson and Jürgen Rehm provides an overview of the history of alcohol’s burden of disease. (pp. 122–127)

## USING SURVEYS TO CALCULATE DISABILITY-ADJUSTED LIFE-YEARS

To quantify the total burden of morbidity attributable to alcohol use, so-called disability weights (DWs) must be generated. General-population surveys can be used to derive DWs from health valuation tasks. This article by Drs. Wolfgang Weidermann and Ulrich Frick discusses the application of three psychometric methods—pairwise comparisons, ranking tasks, and visual analog scales—in general-population surveys and outlines their strengths and weaknesses. In addition, the article discusses a recently proposed health valuation framework, which highlights the underlying cognitive processes from a social judgment perspective. Furthermore, it presents a structured data-collection procedure that seems promising in deriving DWs from general-population surveys. (pp. 128–134)

## ASSESSING THE IMPACT OF ALCOHOL USE ON COMMUNITIES

Community indicators are used to assess the impact of alcohol on communities. For communities, indicator data can be used to inform priority-setting agendas by identifying specific concerns within a community, guide policy and education initiatives, monitor community status on a particular measure over time or in comparison with other communities, and evaluate programs or policies. This article by Drs. Andrea Flynn and Samantha Wells reviews the main data sources for community indicators, discusses their strengths and limitations, and discusses indicators used in reference to four main topics relating to alcohol use and problems at the community level: alcohol use, patterns, and problems; alcohol availability; alcohol-related health outcomes/trauma; and alcohol-related crime and enforcement. (pp. 135–149)

## FOCUS ON: TRAUMA AND EMERGENCY OUTCOMES

Hospital emergency departments (EDs) often see patients arriving with alcohol-attributable injuries. For this reason, researchers frequently use EDs to examine the relationship between alcohol consumption and injury risk using different study designs. As reported by Dr. Cheryl J. Cherpitel, these analyses found elevated injury risk after alcohol consumption, with the extent of risk increase depending on factors such as drinking patterns, concurrent use of other drugs, or even study design. Additionally, Dr. Cherpitel explores numerous other aspects of the relationship between alcohol use and injury risk assessed by ED studies. (pp. 150–154)

## FOCUS ON: CHRONIC DISEASES AND CONDITIONS RELATED TO ALCOHOL USE

Numerous chronic diseases and conditions are entirely or partially attributable to heavy alcohol use; for other conditions, however, alcohol can have a beneficial effect. Various factors, such as the average amount and pattern of alcohol consumption, have been found to influence alcohol’s impact on the mortality and morbidity related to chronic diseases and conditions. However, as Mr. Kevin D. Shield and Drs. Charles Parry and Jürgen Rehm report, although alcohol consumption indisputably contributes to the burden of chronic diseases and conditions, the methods currently used to calculate the relative risks and alcohol-attributable fractions have several limitations. Moreover, new studies and confounders may help further refine our knowledge of which chronic diseases and conditions are causally linked to alcohol consumption. (pp. 155–173)

## FOCUS ON: ALCOHOL AND MORTALITY

Alcohol consumption has long been recognized as a risk factor for mortality. By combining data on alcohol per capita consumption, alcohol-drinking status and alcohol-drinking patterns, risk relationships, and mortality, the Comparative Risk Assessment Study estimated alcohol-attributable mortality for 1990 and 2010. In this article, Drs. Jürgen Rehm and Kevin D. Shield discuss alcohol’s role in the global burden of mortality. (pp. 174–183)

## FOCUS ON CHILDREN AND PREADOLESCENTS

Because there are few surveillance studies of alcohol use and alcohol-related problems among children and preadolescents, estimating the alcohol burden in this population is especially difficult. This article by Dr. John E. Donovan summarizes information from U.S. national and Statewide surveys on the prevalence of alcohol use among children in grades 6 and lower. Although the rates of alcohol use are relatively low in this population, substantial numbers of children do in fact have experience with alcohol. Limited available data highlight the need for better ongoing surveillance of this population. Although alcohol burden in children appears relatively low, it is increased through the alcohol use and abuse of their parents, and through the increased likelihood among early drinkers of alcohol problems and other negative outcomes in adolescence and young adulthood. (pp. 186–192)

## PREVALENCE AND PREDICTORS OF ADOLESCENT ALCOHOL USE AND BINGE DRINKING IN THE UNITED STATES

The Monitoring the Future study is an annual survey among 8th-, 10th-, and 12th-grade students assessing their alcohol use, thereby allowing researchers to better understand adolescents’ consumption patterns during this vulnerable developmental period. These surveys have found high prevalence of drinking and binge drinking by the time students leave high school, report Drs. Megan E. Patrick and John E. Schulenberg. The authors also discuss the factors that influence adolescent alcohol use, such as parent and peer relationships, school and work, behavioral and drug-use problems, or personality characteristics, as well as review potential long-term effects of adolescent alcohol consumption. (pp. 193–200)

## EXCESSIVE ALCOHOL CONSUMPTION AND RELATED CONSEQUENCES AMONG COLLEGE STUDENTS

Research shows that multiple factors influence college drinking, from an individual’s genetic susceptibility to the positive and negative effects of alcohol, alcohol use during high school, campus norms related to drinking, expectations regarding the benefits and detrimental effects of drinking, penalties for underage drinking, parental attitudes about drinking while at college, whether one is member of a Greek organization or involved in athletics, and conditions within the larger community that determine how accessible and affordable alcohol is. This article by Drs. Aaron White and Ralph Hingson examines recent findings about the causes and consequences of excessive drinking among college students relative to their noncollege peers and many of the strategies used to collect and analyze relevant data, as well as the inherent hurdles and limitations of such strategies. (pp. 201–218)

## FOCUS ON: WOMEN AND THE COSTS OF ALCOHOL USE

Although there are proven beneficial effects associated with light-to-moderate drinking, such levels of drinking also are associated with increased risks of breast cancer and liver problems, and heavy drinking increases risks of hypertension and bone fractures and injuries. In addition, women’s heavy-drinking patterns and alcohol use disorders are associated with increased likelihood of many psychiatric problems, including depression, posttraumatic stress disorder, eating disorders, and suicidality, as well as increased risks of intimate partner violence and sexual assault. In this article, Drs. Sharon C. Wilsnack and Richard W. Wilsnack and Ms. Lori Wolfgang Kantor discuss drinking patterns among women during midlife and the risks of heavy drinking in. this population. (pp. 219–228)

## FOCUS ON: ETHNICITY AND THE SOCIAL AND HEALTH HARMS FROM DRINKING

Native Americans, Hispanics, and Blacks are at higher risk of experiencing alcohol-attributable harms than Whites or Asians. This article by Drs. Karen G. Chartier, Patrice A.C. Vaeth, and Raul Caetano examines this health disparity with regard to alcohol and unintentional injuries, intentional injuries, fetal alcohol syndrome, gastrointestinal diseases, cardiovascular diseases, cancers, diabetes, and infectious diseases. (pp. 229–237)

## GAPS IN CLINICAL PREVENTION AND TREATMENT FOR ALCOHOL USE DISORDERS

Numerous prevention and treatment approaches have been developed to ameliorate the morbidity, premature mortality, and other social and economic burdens on society caused by heavy drinking. Those measures can be targeted at different groups of drinkers, including nondependent heavy drinkers, those with functional dependence, and those with alcohol use disorders. However, as Dr. Mark L. Willenbring explains, the impact of current selective prevention and treatment strategies on public health is unclear. For example, existing screening and brief interventions for nondependent drinkers may potentially have a large public health impact, whereas more effective ways are needed to reduce the public health burden associated with functional dependence and alcohol use disorders. (pp. 238–243)

## THE WORLD HEALTH ORGANIZATION’S GLOBAL MONITORING SYSTEM ON ALCOHOL AND HEALTH

With growing awareness of the impact of alcohol consumption on global health, the demand for global information on alcohol consumption and alcohol-attributable and alcohol-related harm as well as related policy responses has increased significantly. This article by Drs. Vladimir Poznyak and Alexandra Fleischmann, Mr. Dag Rekve, Ms. Margaret Rylett, and Drs. Jürgen Rehm and Gerhard Gmel examines the increasing demand from World Health Organization (WHO) Member States and the latest policies set forth by the WHO in response to alcohol’s growing burden of disease worldwide. (pp. 244–249)

## RESULTS FROM THE NIAAA EXPERT PANEL ON ALCOHOL AND CHRONIC DISEASE EPIDEMIOLOGY

NIAAA Expert Panel on Alcohol and Chronic Disease Epidemiology workshop provided an excellent forum for summarizing the current state of the field of alcohol research and for identifying future research opportunities. This article by Drs. Rosalind A. Breslow and Kenneth J. Mukamal reviews the outcomes of the workshop and the ideas that highlight areas in need of additional study and offer a roadmap for moving forward across a variety of methodological approaches and content areas. (pp. 250–259)

